# Microcrystalline Cellulose—A Green Alternative to Conventional Soil Stabilizers

**DOI:** 10.3390/polym16142043

**Published:** 2024-07-17

**Authors:** Lazar Arun, Evangelin Ramani Sujatha, Jair Arrieta Baldovino, Yamid E. Nuñez de la Rosa

**Affiliations:** 1 Centre for Advanced Research in Environment, School of Civil Engineering, SASTRA Deemed to be University, Thanjavur 613401, Tamil Nadu, India; arunlazar1993@gmail.com; 2 Applied Geotechnical Research Group, Department of Civil Engineering, Universidad de Cartagena, Cartagena de Indias 130015, Colombia; jarrietab2@unicartagena.edu.co; 3 Faculty of Engineering and Basic Sciences, Fundación Universitaria Los Libertadores, Bogotá 1112211, Colombia

**Keywords:** kaolin, biopolymer, microcrystalline cellulose, compaction behavior, deformation behavior, UCS

## Abstract

Biopolymers are polymers of natural origin and are environmentally friendly, carbon neutral and less energy-intense additives that can be used for various geotechnical applications. Biopolymers like xanthan gum, carrageenan, chitosan, agar, gellan gum and gelatin have shown potential for improving subgrade strength, erosion resistance, and as canal liners and in slope stabilization. But minimal research has been carried out on cellulose-based biopolymers, particularly microcrystalline cellulose (MCC), for their application in geotechnical and geo-environmental engineering. In this study, the effect of MCC on select geotechnical properties of kaolin, a weak, highly compressible clay soil, like its liquid and plastic limits, compaction behavior, deformation behavior, unconfined compression strength (UCS) and aging, was investigated. MCC was used in dosages of 0.5, 1.0, 1.5 and 2% of the dry weight of the soil, and the dry mixing method was adopted for sample preparation. The results show that the liquid limit increased marginally by 11% but the plasticity index was nearly 74% higher than that of untreated kaolin. MCC rendered the treated soil stiffer, which is reflected in the deformation modulus, which increased with both dosage and age of the treated sample. The UCS of kaolin increased with dosage and curing period. The maximum UCS was observed for a dosage of 2% MCC at a 90-day curing period. The increase in stiffness and strength of the treated kaolin with aging points out that MCC can be a potential soil stabilizer.

## 1. Introduction

Soil stabilization attempts to enhance the geotechnical properties of soil for various construction needs and ensures optimal performance in marginal soils. Soil stabilization, one of the most commonly adopted techniques to improve soil nature, uses lime, cement, bitumen, fly ash, sodium sulfate and other various additives to modify soil properties to meet the design criteria of a construction project. The choice of the additive depends on the target strength, economic viability, project size and environmental considerations [1]. In recent times, phenomena like climate change, global warming, and their aftereffects, like sea level rise, frequent natural disasters, and extreme precipitation, underline the need for the choice of materials that are more environmentally friendly, less energy-intensive in production, and minimally resource intensive. However, conventional stabilizers, particularly calcium-based stabilizers like lime and cement, do not adhere to these preferable requirements [2,3]. These synthetic stabilizers lead to the emission of greenhouse gases (GHG) that are responsible for global warming in their production stage and alter the soil environment, permanently causing soil pollution [2,4]. Cement production in the years between 2010 and 2018 increased from 3310 to 4100 million tons, which is nearly an increase of 23.8%, contributing a significant 8% of total carbon dioxide emissions globally [3,4]. The European Union Commission has set a target to cut GHG by 85% by the year 2050 in comparison to the year 1990 [5,6]. The need to find alternative environmentally friendly soil stabilizers is particularly important as large quantities of stabilizers are commonly required for improving soil properties. The recommended soil stabilizer should not only satisfy the environmental requirements but also show a satisfactory improvement in the required geotechnical properties, exhibit durability, and should also be an economical alternative. One widely investigated alternative in the last decade has been biogenic materials—materials of biological origin. A few techniques that have been widely researched for their potential in ground improvement and modification are the use of microbes, enzymes and biopolymers. Biopolymers have shown great potential in improving and modifying the soil properties for various geotechnical and geo-environmental applications like strengthening foundation soil, improving subgrade strength, stabilizing slopes and modifying soil and as contaminant barriers, clay liners and canal liners.

Biopolymers are polymers of natural origin and are produced/extracted from plants, animals and micro-organisms through various techniques. Biopolymers have a wide range of applications in the construction industry, like ceramics, tile adhesives and drill fluids [7,8]. In the field of geotechnical engineering, biopolymers improve the properties of marginal soils [3]. Biopolymers like xanthan gum, guar gum, gellan gum, chitosan, casein, carrageenan, gelatin, β glucan have been examined for their potential to modify the soil properties. Xanthan gum, one of the first biopolymers to be comprehensively investigated as a soil stabilizer, increased the strength of clay by nearly 470% [9]. Xanthan gum also effectively improved the unconfined compressive strength (UCS) of bentonite and kaolin by the formation of cation bridges and hydrogen bonds, allowing the monomers to interact with the electrically charged surface of the clay particles [10]. Similarly, biopolymers gellan gum and agar contributed to strength gain by 114.8% and 52%, respectively, at a 1% dosage [11]. Reference [12] used xanthan gum and carrageenan to improve the strength of kaolin and reported that 0.5% carrageenan showed better performance than xanthan gum. In the study in Reference [13], sodium alginate was used to improve the strength of highly compressible clay by two methods, spraying and soaking, and it was found that the UCS increased significantly in the spraying method. In the soaking method, the UCS increased at a 1-day curing period, but on further aging, the UCS decreased. In the study in Reference [14], β glucan was used to enhance the cohesion in lean clay, and it was found that 2% β glucan improved cohesion by 8.38 times owing to the ionic bond formed between a double layer of water, anionic β glucan and clay particles, which are negatively charged. In the study in Reference [15], carboxymethyl cellulose was used to stabilize low-compressible silt, and an increase of 9.34 times in strength at a 1% biopolymer dosage was observed. In the study in Reference [16], the mechanical stability of soils treated with chitosan was investigated and it was found that chitosan with a higher molecular mass had positive effects on the UCS of treated soil compared to chitosan with a lower molecular mass. Chitosan has also been used to remove contaminants from soil [17].

This study explores the possibility of using a lesser investigated biopolymer, microcrystalline cellulose (MCC), to improve the geotechnical properties of kaolin, a low-swelling clay. MCC, a form of cellulose, is not only minimally investigated for geotechnical applications but is also abundantly available. Select geotechnical properties like the liquid and plastic limits, compaction behavior, deformation behavior and unconfined compressive strength are investigated for various dosages of MCC varying from 0.5% to 2% of the dry weight of soil. The effect of aging on the strength of the MCC-treated kaolin is studied for a period of 90 days. The results of this study indicate that MCC is a promising biopolymer that can be used for various geotechnical applications where strength improvement is a major criterion, like improving the strength of soil for foundations, subgrade of highways and airports and stabilizing slopes.

## 2. Materials and Methods

### 2.1. Soil

**Figure 1:**
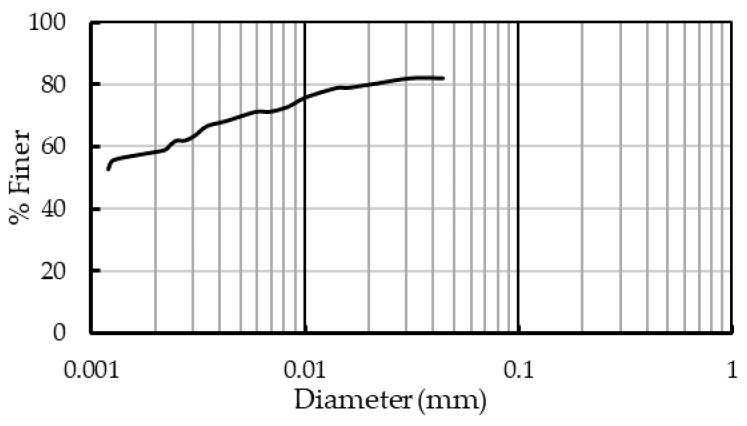
Gradation curve of kaolin.

Commercially available kaolin, a low-swelling and high-plastic clay, was used for the study was procured from M/s Aastra Chemicals, Chennai, India. The hydrometer analysis of the soil showed that nearly 59% of the particles were clay-sized, i.e., below 0.002 mm, while 23% fell into the silt size, i.e., between 0.075 mm and 0.002 mm (Figure 1).

The clay, kaolin, has a specific gravity of 2.62 and an organic content of less than 1%. The select geotechnical properties of kaolin are listed in Table 1.

**Figure 2:**
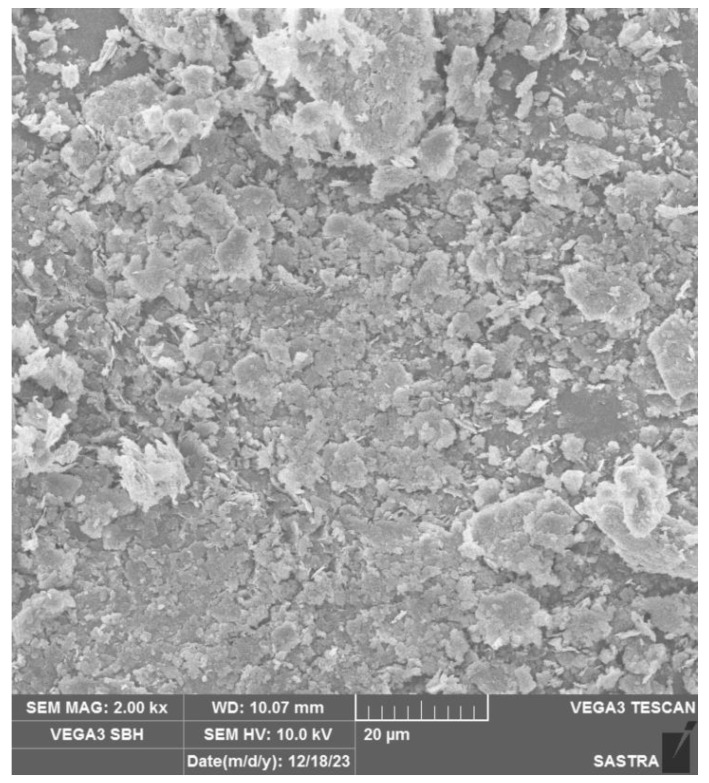
SEM micrograph of kaolin.

The soil, kaolin, is classified as a highly compressible clay based on the unified soil classification system. The clay at its OMC was in soft consistency and exhibited a low strength, indicating the need for improvement to achieve the target strength of 200 kPa after 28 days. However, the soil satisfied the permeability requirement of a minimum of 10^−7^ m/s for use as a contaminant barrier. Also, no desiccation cracking was observed on the surface of the shrinkage pats, indicating its suitability as a contaminant barrier. The micrograph from the SEM (Figure 2) shows the surface morphology of kaolin exhibiting a crystalline structure. The micrograph shows flaky clay particles, i.e., thin platelets with a high aspect ratio. The crystalline nature of kaolin impacts its rheological behavior. 

### 2.2. Microcrystalline Cellulose (MCC)

**Figure 3:**
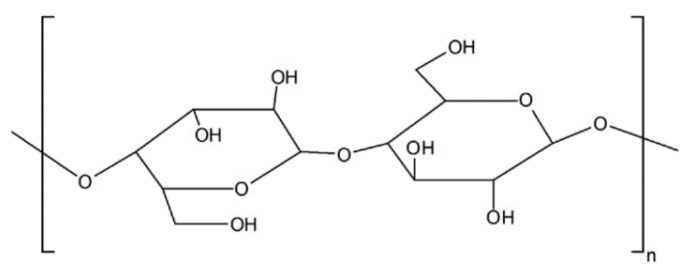
Molecular structure of MCC [20].

MCC (C_14_H_26_O_11_)n is a polysaccharide with glucose units and is the primary constituent in plant fibers. It is white in color and a free-flowing powder that has excellent water absorptive, swelling and dispersion properties that make it different from alpha cellulose. It is a pure form of spray-dried cellulose powder derived from wood pulp and is free from both organic and inorganic contaminants. It is chemically inert and insoluble in water, a dilute acid, a common organic solvent, or oil but is partially soluble with slight swelling in dilute alkali. It finds various applications like an anti-caking agent, emulsifier, fat substitute and bulking agent in the food and cosmetic industry. It is also widely used in the pharmaceutical and medical industry owing to its excellent compressibility. Its properties, like large surface area, high strength owing to its semi-crystalline fibrillary structure, stiffness, crystallinity, lightness and bio-degradability, make it an attractive alternative for many industrial, geotechnical and geo-environmental applications [18,19]. It also suffers some limitations, like low wettability and incompatibility with other polymeric matrices. MCC is anionic in nature with a pH ranging from 5 to 7.5. Commercially available MCC, procured from M/s Amster Microcell, Gujarat, India, was used for the study. The molecular structure of MCC is shown in Figure 3.

## 3. Experimental Investigation

The soil was sieved through the required sieves for the various experiments, as per the specifications provided in the respective codes. The MCC was dry-mixed thoroughly with the soil as it is not soluble in water, and the required water content was gradually added to the soil. The soil–MCC–water mixture was thoroughly hand-mixed to achieve a homogeneous blend. The liquid- and plastic-limit tests of the soil and soil–MCC mixes were carried out in accordance with the procedure outlined in ASTM D4318 [21]. Soil and soil–MCC mixtures were compacted at the standard compaction effort (ASTM D698) [22]. Cylindrical samples, 38 mm diameter and 76 mm height, were prepared at the respective OMC of the dosage. Initial tests were carried out 6 h after the preparation of the samples, which will be hereafter referred to as 0 days to decide the dosage of the mix. The UCS test was conducted in line with the procedure outlined in ASTM D2166 [23]. Based on the UCS of the soil, the dosage was fixed at 0.5%, 1.0%, 1.5% and 2%. The soil–MCC–water mixes at the said dosages were control-cured by preserving them in polythene air-lock covers for 7, 28, 56 and 90 days to study the effect of aging on the strength of soil–MCC mixes. These mixes were then molded into cylinders for the UCS test on the respective days of curing and were tested to determine the UCS after the respective curing period.

X-ray diffraction (XRD) analysis was carried out using a D8 focus XRD instrument of the Bruker, Ettlingen, Germany, to analyze the changes in chemical composition, if any, and physical properties. Similarly, the Fourier Transform Infra-Red spectrograph of the Perkin Elmer, Waltham, MA, USA was used to identify shifts in molecular groups with MCC addition. Surface morphological changes were delineated using a scanning electron microscope of the TESCAN VEGA 3, Brno, Czech Republic. Soil with a 2% MCC dosage at its respective water content was prepared and dried for XRD, FTIR and SEM analysis. Powder samples were used for XRD and SEM analysis, while dry pellets were prepared for FTIR analysis.

## 4. Results and Discussion

The effect of MCC dosage and aging on the selected geotechnical properties of the selected soil, kaolin, is discussed in the following sections.

### 4.1. Plasticity of MCC-Treated Soil

**Figure 4:**
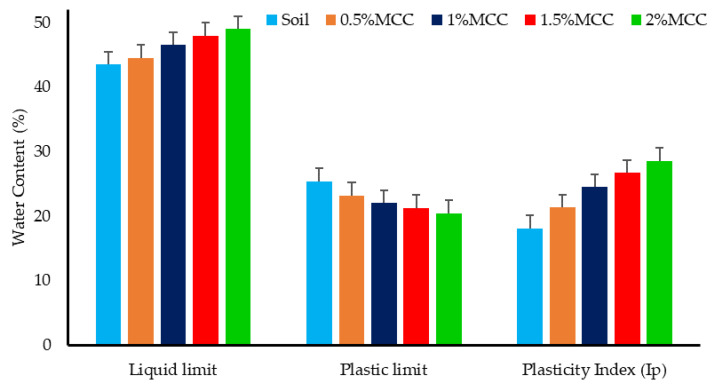
Effect of MCC on the plastic behavior of kaolin.

The liquid and plastic limits of the soil define the soil’s plasticity nature. The liquid limit (LL) is the water content at which the saturated soil possesses a minimum measurable undrained shear strength (su). At LL, the soil su ranges from 1 kPa to 3 kPa, and at water content beyond LL, the soil behaves like a viscous slurry. LL also indicates the soil’s water-holding capacity. The plastic limit (PL) is the threshold water content at which the soil exhibits plastic behavior, and the plasticity index (PI) represents the range of water content over which the soil is plastic. These parameters, LL, PL and PI, are paramount in classifying fine-grained soil. Figure 4 shows the effect of MCC dosage on the LL, PL and PI of biopolymer-modified kaolin. The percentage variation in the three trials conducted to determine LL and PL was limited to 5%.

Kaolin, a low-swelling clay used for the study, has an LL of 44%, and with the addition of MCC, it tended to increase gradually. The increase in LL, though, was marginal and was maximum at 2% MCC, where the LL was 49%, i.e., an increase of nearly 11%. The marginal increase in LL can be attributed to the hygroscopic nature of the soil and the anionic nature of the MCC, which tends to increase the cation exchange capacity of kaolin and promote flocculation [24]. The tendency of MCC to promote flocculation in the soil–MCC mixes increased the resistance to the workability of the mixes and decreased the PL marginally (Figure 4). The plastic limit of the soil was 27.6%, and with the addition of MCC, it decreased to 20.5% at 2% MCC addition. The change in PL was smaller compared to the change in LL with the addition of MCC, and thereby, the PI increased significantly with MCC dosage, indicating that the range over which the soil remained plastic increased with MCC addition.

The PI of kaolin was 16.4%, while the PI of the soil–MCC mix with the maximum investigated MCC dosage of 2% was 28.5%, i.e., a nearly 74% increase was observed. The significant increase in PI indicates that the addition of MCC increases the plastic nature of the soil, and this can be attributed to the hydrophilic nature of MCC.

### 4.2. Compaction Behavior of MCC-Treated Soil

**Figure 5:**
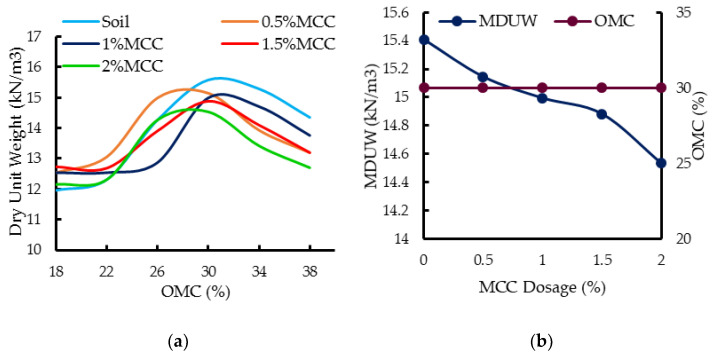
(**a**) Effect of MCC on compaction curves. (**b**) Effect of MCC on MDUW and OMC.

The compaction behavior of the treated soil decides its suitability for various geotechnical and geo-environmental applications. The nature of the compaction curves of both soil and soil with MCC were similar, showing a marginal change in MDUW with a change in water content (Figure 5a), indicating that the addition of water had a lesser effect on the dry unit weight of the treated soil. The addition of MCC did not appreciably modify the MDUW of the soil (Figure 5b), and the OMC of both soil and soil–MCC mixes remained the same at 30% for all the investigated dosages. The MDUW of soil was 15.41 kN/m^3^, while that of 2% soil + MCC mix was 14.53 kN/m^3^, i.e., MDUW decreased by nearly 5.7%. The marginal reduction in MDUW can be attributed to the resistance offered by the soil–MCC mix owing to the lesser particle-to-particle interaction caused by MCC addition, as also observed in the case of carboxymethyl cellulose [25]. The nature of MCC in promoting compressibility can also contribute to a lower MDUW. Also, both kaolin and MCC carry negative charges at the surface, which can lead to particle dispersion and lesser packing, eventually leading to a lower MDUW. 

MCC are quasi-neutral particles. Though hydrophilic, they can stabilize the oil/water interface, and at times, their crystal planes can expose a hydrophobic surface [26,27]. This could be the reason for the constant OMC despite the addition of MCC at various dosages. Also, the added dosage and increment in dosage were very minimal, and thereby, the OMC may not have changed.

### 4.3. Deformation Behavior and Strength of MCC-Treated Soil

**Figure 6:**
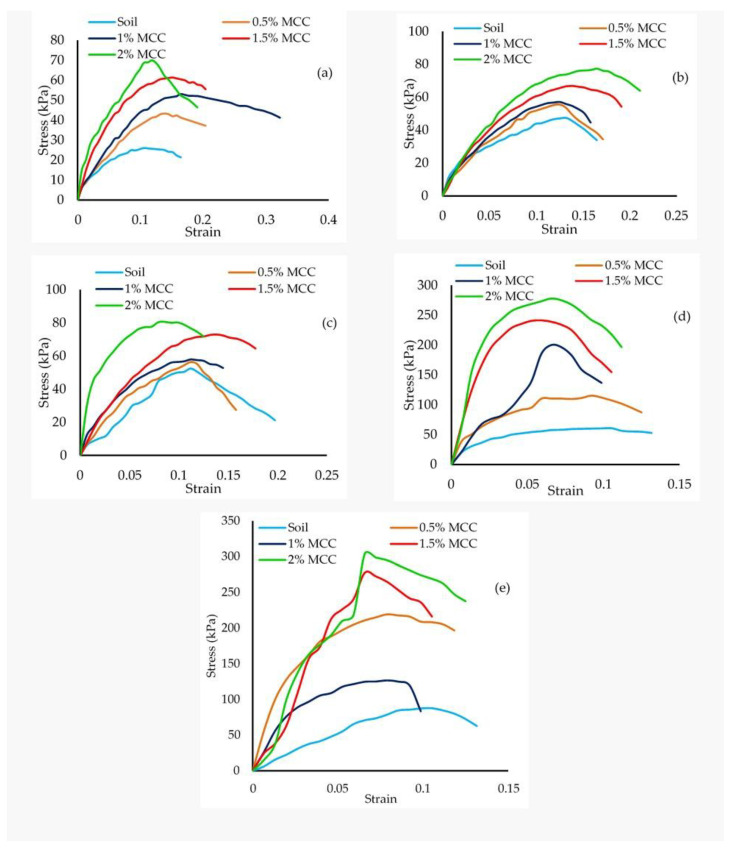
Stress–strain response of MCC-treated soil. (**a**) 1 day, (**b**) 7 day, (**c**) 28 day, (**d**) 56 day and (**e**) 90 day.

The stress–strain response of the soil reflects its deformation behavior on loading. Figure 6a–e show the stress–strain response of kaolin treated with the selected dosages of MCC at a curing period of 1, 7, 28, 56 and 90 days. The resistance to load increased with an increase in the MCC dosage and curing period. Though the increase in dosage showed a marked increase in higher resistance to load, the failure strain did not exhibit a clear trend. The nature of the deformation curves at the investigated dosages exhibited a similar trend up to a 28-day curing period, with a gradual increase in resistance to load, but at the 56- and 90-day curing period, there was a marked increase in resistance to load. Failure strain tended to decrease with aging. For example, a maximum failure strain of 11.84% was observed at the 1-day curing period, and a minimum of 6.58% was observed at the 90-day curing period (Figure 6b,e) at a dosage of 2%. The deformation curves for higher dosages show clear peaks at the 90-day curing period, which was distinctly different from the deformation behavior observed at other curing periods, indicating the stiffening in the soil matrix with aging, particularly at the higher dosages of 1.5 and 2%. The stiffening may also be attributed to the formation of cementitious compounds.

**Figure 7:**
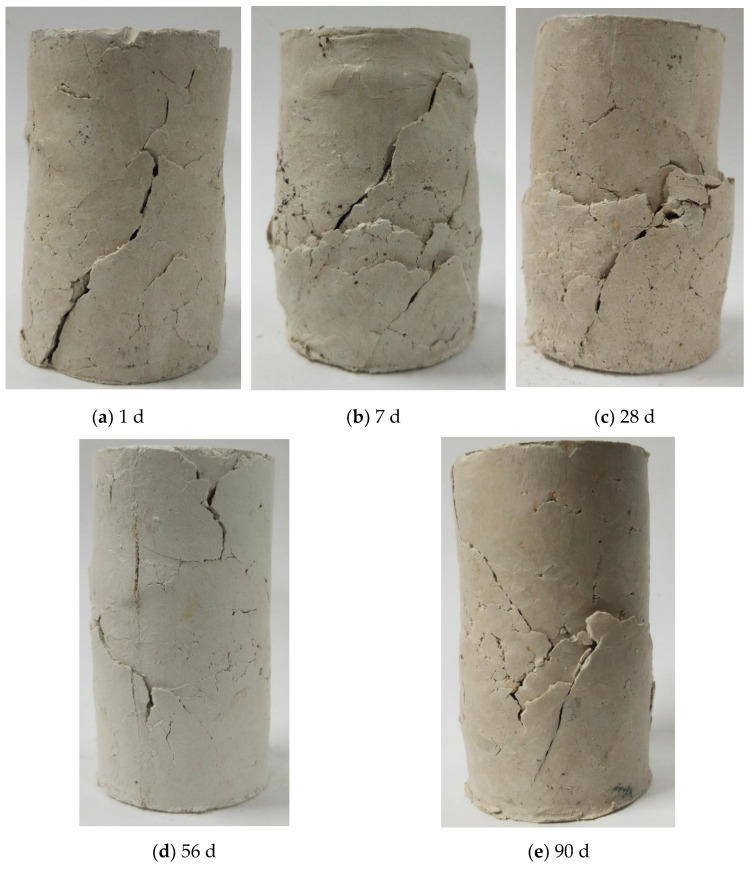
Effect of aging on the 2% MCC-treated kaolin.

Aging tended to decrease the plastic nature of the soil as observed from the mode of failure in the treated samples, as seen in Figure 7, and from the figure it can be observed that the samples failed with multiple cracks and pronounced bulging upto 28 day but at higher curing periods, the samples did not bulge at failure, indicating the increased stiffness of the soil matrix. The increase in inter-particle bonding as a result of hydrogen bonds formed because of the presence of biopolymer could have contributed to the change in load resisting capacity of the treated kaolin. A comparison between cement- and lime-treated highly compressible clay [28] and 2% MCC-treated kaolin, which is also highly compressible is made in Table 2.

The deformation modulus, defined as the ratio of peak stress to corresponding strain, is a measure of strength and increased with both dosage and aging (Table 3). At the 1-day curing period, the deformation modulus increased by 2.42 times for the highest investigated dosage of 2% MCC, while at a 90-day curing period for the same dosage, the deformation modulus significantly increased by 5.31 times when compared with untreated kaolin. The improvement in the resistance to higher stresses in MCC-treated soil can be attributed to the tendency of MCC to form hydrogen bonds as MCC has a hydroxyl (OH^−^) on the edges (Figure 3), which is both a hydrogen bond donor and acceptor, but mostly a donor [3]. A large number of sites were available in the biopolymer for hydrogen bonds to form, and these large numbers made the hydrogen bonds stronger in biopolymer-treated soil, leading to higher resistance to loads. The increased deformation modulus indicates increased stiffness in treated kaolin, which points to electrosteric stabilization of the biopolymer MCC. Also, electrosteric stabilization can cause bio-filling between the soil particles [29], and this void filling results in a denser matrix that offers higher resistance to loads.

**Figure 8:**
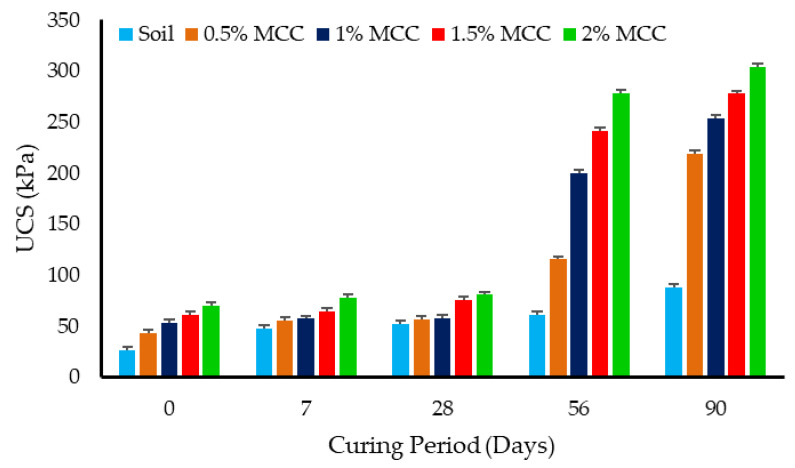
Effect of MCC dosage and aging on the UCS of treated kaolin.

The UCS increased with an increase in dosage and aging in MCC-treated soil specimens, as observed in Figure 8. The percentage variation between the investigated samples to determine the UCS at each dosage and curing period was limited to 3%. At the 1-day curing period, the UCC of kaolin was 26 kPa, while that of kaolin treated with 2% MCC was nearly 70 kPa, recording an increase of approximately 2.69 times the strength of untreated kaolin. The highest rate of increase was observed at a 0.5% MCC dosage, where the UCS increased by 1.66 times, and then with the further addition of MCC in dosages of a 0.5% increment, gradually increased to 70 kPa. Also, with aging, there was a gradual and marginal increase in strength for all investigated dosages up to a curing period of 28 days, but at the longer curing period of 56 and 90 days, there was a marked increase in strength. There was an approximate increase of 1.15 times in the UCS of 2% MCC-treated kaolin at the end of the 28-day curing period, while the UCS markedly increased by 4.34 times at the end of the 90-day curing period compared with the UCS at the 1-day curing period. Also, it can be observed that the rate of increase in the UCS between the 56- and 90-day curing periods was 1.09 times, while that between the 28- and 56-day curing period was 3.45 times, indicating that despite the increase in strength, the rate of increase diminished with further time.

**Figure 9:**
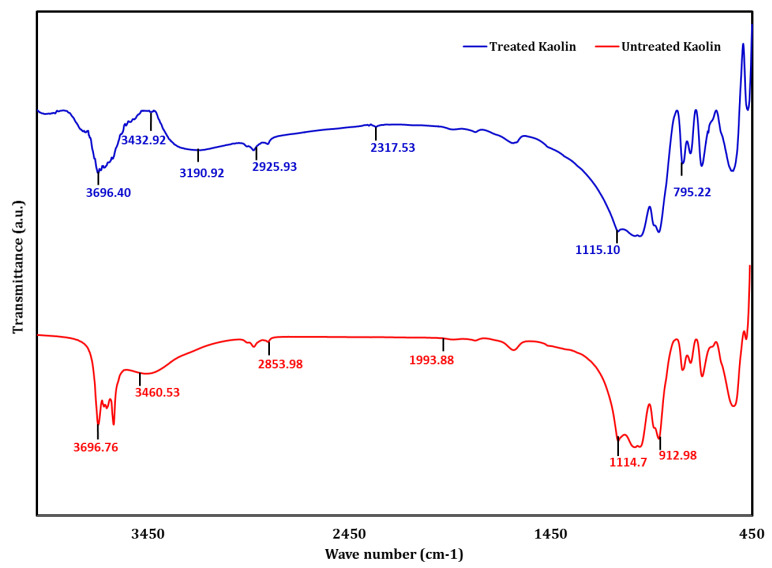
FTIR spectrum for koalin and MCC-treated kaolin.

The available sites for the formation of hydrogen bonds increased with an increase in the MCC dosage, and this increase can be the reason for the increase in the UCS of the treated kaolin. The hydroxyl (OH–) available on the edges of the biopolymer (Figure 3) caused surface polarization, leading to water adsorption and void filling that resulted in an increase in the strength of the treated soil [3]. Water adsorption bonds in the regions 3460.53 cm^−1^ to 2924.60 cm^−1^ [29], as seen from Figure 9 in the FTIR spectra, suggested O-H stretching. Also, H-bonded OH groups were noticed at wavelengths 3696.40 cm^−1^ to 3190.92 cm^−1^ [30], and this is indicative of hydrogen bonding in treated kaolin as a result of biopolymer MCC addition. This hydrogen bonding in treated kaolin can be attributed to the increase in the UCS of the treated kaolin. The broad peak around 3432.93 cm^−1^ corresponded to O-H stretching vibrations of adsorbed molecules and also indicated hydrogen bonding, which was a primary reason for increased strength and stiffness in treated kaolin. The broad peaks between 3200 and 3600 in treated kaolin were also indicative of hydrogen bonding. The strong peaks around 1031.22 cm^−1^ to 1007.24 cm^−1^ in both treated and untreated kaolin indicated the presence of a Si-OH vibration [30]. The bands with wavenumbers around 3538.85 cm^−1^ to 3432.92 cm^−1^ also denoted the Si-OH vibrations in the clay minerals present in the soil. The wavelengths 2352.34 cm^−1^ to 2317.53 cm^−1^ indicated the stretching vibrations in C=O and C=C groups [31] in treated soil, which was a result of the combination of kaolin and MCC. The peaks 56.675 and 56.56 suggested the presence of crystalline C-S-H, which may help in increasing the interlocking between the soil particles and act as a mechanical reinforcement, which, in turn, increased the shear strength by providing resistance against sliding and deformation. The presence of these C-S-H crystals filled voids, lowered permeability and densified the soil matrix. The same peaks, 56.675 and 56.56, were also indicative of calcium aluminate phases (JCPDS Number: 33-0302), which may also lead to pore filling with water and the formation of hydration products, like calcium aluminum hydrates (C-A-H) and calcium aluminum silicate hydrate (C-A-S-H), which can act as binding agents that improve the strength, increase stiffness and result in matrix densification.

**Figure 10:**
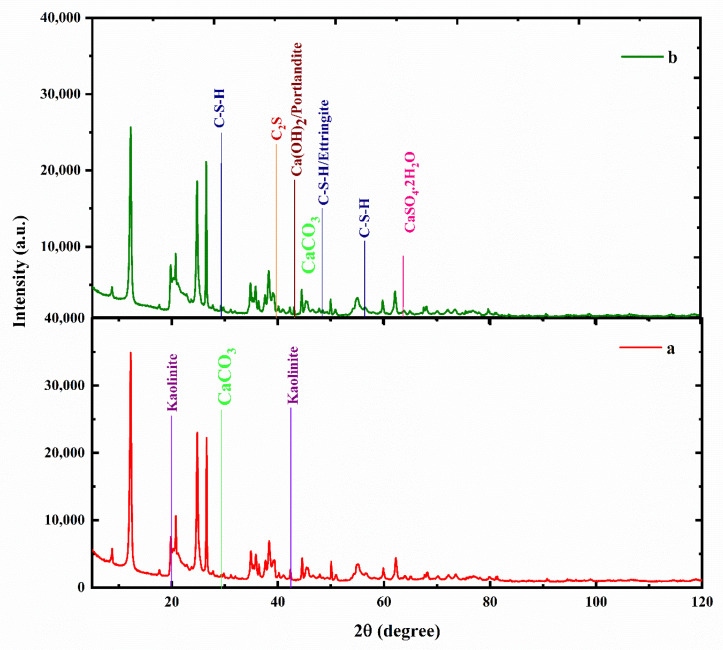
XRD diffractogram of (**a**) kaolin and (**b**) MCC-treated aolin.

The XRD diffractogram (Figure 10a) shows the presence of kaolinite (Al_2_(Si_2_O_5_) (OH)_4_) as the predominant phase in the clay with a trace of quartz (SiO_2_) and zeolite (SiO_2_), with percentages of 73%, 23% and 1%, respectively. The peaks at 42.3° in the diffractograms (Figure 10a,b) signified the presence of kaolin in both kaolin and MCC-treated kaolin, while the peak at 43.11° in treated kaolin showed abundant forms of calcite (CaCO_3_) in treated kaolin. The presence of calcite suggests the scope for the formation of cementitious material with the addition of MCC, as kaolin will remain inert in the absence of an additional calcium source. The XRD diffractogram for MCC-treated kaolin (Figure 10b) exhibited peaks that corresponded to various forms of calcium silicate hydrates (29.285°, 29.329° and 29.784°) corresponding to JCPDS numbers 03-0647 and 31-0301. Also, the peaks 47.094, 47.142, 47.871, 48.349 and 48.524 were indicative of the presence of various phases of C-S-H (JCPDS number 31-0301) and also overlapped with the peak for ettringite (JCPDS number 41-1451). These observations from the XRD diffractogram suggest that the strength gain can be attributed to the formation of cementitious material C-S-H that acts as a binding/cementing agent. Peaks 47.094, 47.142, 47.871, 48.349, 48.524, 56.675 and 56.56 were also suggestive of ettringite, which is also a strong binding agent.

**Figure 11:**
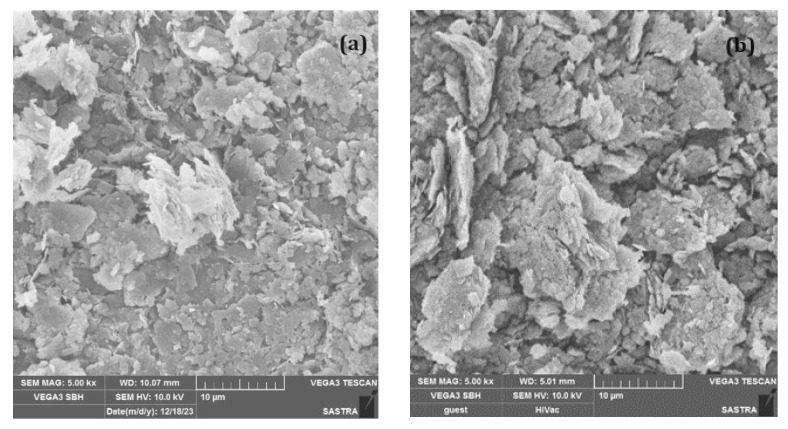
SEM micrographs. (**a**) kaolin and (**b**) MCC-treated kaolin.

The surface morphology of the kaolin treated with MCC in the form of micrographs using SEM is presented in Figure 11a,b. The surface morphology of kaolin, as observed in Figure 11a, was flaky and layered with different particle sizes. The kaolin structure had a size of 1–10 μm with the number of sheets per layer in the range of 10 to 50. It is clear that there was a large quantity of quartz mineral, which is an impurity present in the kaolin mineral, as seen in Figure 11a [30].

The surface morphology of treated kaolin is also flaky and layered. Though similar to untreated kaolin, it shows a pronounced, well-defined and abundant crystalline nature (Figure 11b) owing to the crystalline nature of MCC. The surface of the particles appeared rough and irregular which could be an indication of high surface area. The rough texture and some inter-particle spaces can be attributed to the water-retention nature of the biopolymer. The layered appearance also suggests effective coating of biopolymer and binding. The matrix of the treated kaolin is denser as the voids are filled by cementitious material C-S-H and ettringite as observed from the XRD (Figure 10b). The void filling can also be a result of the electrosteric stabilization. Hydrogen bonding and electrosteric can synergetically improve the mechanical properties of treated kaolin.

## 5. Conclusions

Biopolymers have been widely researched for their use as soil stabilizers to improve various geotechnical properties of the soil in the last decade, attempting to replace conventional stabilizers like lime and cement. In this study, a lesser investigated biopolymer for soil stabilization, microcrystalline cellulose, was examined for its choice as a soil stabilizer. The results of the study showed that MCC impacted LL marginally by 11% and PI markedly by nearly 74% at the maximum investigated dosage of 2% MCC. The MDUW of treated kaolin was reduced minimally by approximately 5.7% at 2% MCC addition, while the OMC remained constant for all dosages of MCC. MCC also rendered the selected clay, kaolin, stiffer, as reflected by the significantly improved deformation modulus. The UCS of kaolin treated with MCC also increased by 2.69 and 3.45 times compared to untreated kaolin at the end of the 1- and 90-day curing periods. The study also showed that the UCS increased with both dosage and time, indicating that the effect of MCC did not diminish with time, which is an important factor to be considered in its choice as a soil stabilizer. The increase in strength of the treated kaolin is primarily attributed to the formation of hydrogen bonding between the biopolymer and kaolin and electrosteric stabilization. The study advocates the choice of MCC as a soil stabilizer to improve the strength of soil not only based on its effect on geotechnical properties of treated soil, particularly strength, but also because it is a natural material obtained from plants, abundantly available in nature and does not adversely affect the soil environment providing a sustainable solution in lieu of conventional stabilizers that cause soil pollution. This study investigated the effect of MCC on select geotechnical properties, but there is further scope to investigate the effect of MCC on long-term aging, compressibility behavior and permeability of treated soil.

## Figures and Tables

**Table 1:** Geotechnical properties of kaolin.

Geotechnical Properties	Value
Liquid limit (%)	52
Plasticity Index (%)	24
Optimum moisture content (OMC, %)	30
Maximum dry unit weight (MDUW, kN/m^3^)	15.5
Unconfined compressive strength (UCS, kN/m^2^)	26
Co-efficient of permeability (k, m/s)	2 × 10^−9^

**Table 2:** A comparison between stabilization of highly compressible clay using cement and lime to 2% MCC-stabilized kaolin.

Soil Properties	Soil (CH)		6% Cement Stabilization	6% Lime Stabilization	2% MCC Stabilization
	From Literature [28]	Current Study	From Literature [28]	From Literature [28]	Current Study
OMC (%)	17	30	18	20	30
MDU (kN/m^3^)	15.40	15.5	14.6	14	14.53
UCS at 1 d (kN/m^2^)	413	26	1654	517	70

**Table 3:** Effect of MCC dosage and aging of MCC-treated kaolin on the deformation modulus.

Deformation Modulus (kPa)					
Curing Period	Soil	0.5% MCC	1.0% MCC	1.5% MCC	2.0% MCC
1	314	413	438	542	760
7	471	576	592	637	808
28	599	644	662	695	1169
56	732	1563	3694	4915	5130
90	1056	3420	3952	5122	5609

## Data Availability

All the data relevant to the manuscript are presented in the manuscript.
